# Supplementary material for: Research on the development and differentiation of the physical literacy scale for Chinese college students
Source: PLoS One. 2026 May 8;21(5):e0348776. doi: 10.1371/journal.pone.0348776 (PMC13155614; doi:10.1371/journal.pone.0348776)
Supplement: Attachment — Submitted filename: Formal reviewer report_PONE-D-25-65782.docx [file pone.0348776.s003.docx]

This manuscript presents the development and validation of a Physical Literacy Scale for Chinese college students using Delphi methodology, item analysis, and exploratory factor analysis (EFA). The topic is relevant and contributes to the growing field of physical literacy assessment in higher education populations.

The study demonstrates methodological effort and large sample size (n = 3,077), which strengthens statistical robustness. Other strengths of this manuscript include a systematic scale development process and a good statistical screening steps. However, several areas require clarification and refinement, particularly in methodological transparency, statistical justification and in the theoretical framing.

The authors can refer to the following suggestions to strengthen their manuscript:

1. The author should add specific statistical values in the abstract.
2. Ensure all abbreviations are defined at first use (e.g., KMO, Ca, Cr, Cs).
3. The author is advised to define and clarify the theoretical model that underpins the four-dimension structure. Mention if the framework is adapted from existing models or newly conceptualized by the authors.
4. The manuscript requires clear justification of the expert selection criteria. Explain if there was a sample size calculation formula or a predefined consensus threshold in selecting the 16 experts.
5. Multiple screening methods were used in the study namely, Internal item correlation, Cronbach’s Alpha, and EFA. It is good if the author can include the sequence of deletion steps, preferably in a flowchart. Justify the use of PCA instead of the common factor analysis.
6. Authors should justify the cutoffs of 0.9 and 0.2 with proper citation and to explain the reason a 50% threshold was chosen.
7. Provide information on how the extracted 10 factor structure related to the originally mentioned four dimensions.
8. The ethical statement is provided appropriately. However, clarify how the anonymity was maintained in the dataset. Confirm whether participation was voluntary and whether any compensation was provided.
9. The restriction on data availability restricted the reviewer from being able to check on the results and the presentation.
10. Minor language editing is required.
