# Supplementary material for: Research on the development and differentiation of the physical literacy scale for Chinese college students
Source: PLoS One. 2026 May 8;21(5):e0348776. doi: 10.1371/journal.pone.0348776 (PMC13155614; doi:10.1371/journal.pone.0348776)
Supplement: Attachment — Submitted filename: Response to Reviewers.docx [file pone.0348776.s004.docx]

**Point-to-point response**

**I am deeply grateful to the reviewers for your valuable suggestions on my research. I would also like to thank the editor for their assistance throughout this process. I have carefully considered these comments and revised the manuscript accordingly. Below are my responses to each suggestion:**

*1. The author should add specific statistical values in the abstract.*

Some relatively important research findings have been supplemented in the abstract.

*2. Ensure all abbreviations are defined at first use (e.g., KMO, Ca, Cr, Cs).*

Based on the modification suggestions you have proposed, the full forms of these abbreviations have been provided, such as in lines 146, 147, 280, etc.

*3. The author is advised to define and clarify the theoretical model that underpins the four-dimension structure. Mention if the framework is adapted from existing models or newly conceptualized by the authors.*

It has been Supplement in lines 110-112 that the four-dimensional framework for physical literacy referenced the Physical Literacy Evaluation Index System Part 1: Adults and Part 2: Adolescents, rather than being newly constructed. This indicator system model was public released by an official Chinese institution and holds a certain degree of authority within China，It has been widely recognized and referenced by numerous scholars in China.

*4. The manuscript requires clear justification of the expert selection criteria. Explain if there was a sample size calculation formula or a predefined consensus threshold in selecting the 16 experts.*

The additions and modifications have been made according to your suggestions, the criteria for expert selection—including professional fields, regional breadth, and corresponding efficiency, etc—have been outlined in Lines 134–140. The selection of expert numbers also references consensus standards in the field of scale development and includes citations.

*5. Multiple screening methods were used in the study namely, Internal item correlation, Cronbach’s Alpha, and EFA. It is good if the author can include the sequence of deletion steps, preferably in a flowchart. Justify the use of PCA instead of the common factor analysis.*

The entry screening process has been visualized as a flowchart per your suggestion. The image is displayed on page 23, with a reference provided at line 365.

*6. Authors should justify the cutoffs of 0.9 and 0.2 with proper citation and to explain the reason a 50% threshold was chosen.*

We have incorporated your suggestions by adding reference annotations to the numerical standards (0.9, 0.2, 50%, etc.) within the Internal Item Correlation Method on line 259, referencing the commonly accepted consensus standards for this method in China.

*7. Provide information on how the extracted 10 factor structure related to the originally mentioned four dimensions.*

We have added relevant explanations in lines 348-355. These 10 factors belong to the second-order factors.

*8. The ethical statement is provided appropriately. However, clarify how the anonymity was maintained in the dataset. Confirm whether participation was voluntary and whether any compensation was provided.*

We have detailed the ethics statement under the Methods section as requested in the email, and provided supplementary explanations regarding anonymity, voluntariness, and other details, primarily in lines 117-125.

*9. The restriction on data availability restricted the reviewer from being able to check on the results and the presentation.*

Regarding data acquisition, we regret that we are unable to present the complete dataset at this time. As this portion of the data is also relevant to another ongoing research project within our group, we cannot disclose it in its entirety. Once all related studies are completed, we will be able to provide the full dataset.

*10. Minor language editing is required.*

The language in the manuscript has been polished.

**We have carefully implemented the revisions you suggested above. Should you have any further valuable suggestions, please feel free to share them. We will continue to spare no effort in refining this work. Thank you once again for your invaluable assistance with this research.**
