# Supplementary material for: Research on the development and differentiation of the physical literacy scale for Chinese college students
Source: PLoS One. 2026 May 8;21(5):e0348776. doi: 10.1371/journal.pone.0348776 (PMC13155614; doi:10.1371/journal.pone.0348776)
Supplement: Attachment — Submitted filename: Response_to_Reviewers_auresp_2.docx [file pone.0348776.s005.docx]

**Point-to-Point Response**

**Dear Academic Editor/Reviewer,**

**Thank you very much for your recognition of my research. We would also like to express my sincere gratitude for your valuable suggestions on improving the manuscript, which have been instrumental in refining its content. We have carefully addressed each of the proposed revisions, and my detailed responses are provided below.**

*1- Some methodological justifications (e.g., cut-off values, PCA vs. common factor analysis, and expert selection criteria) would benefit from stronger support through international references, not only local or regional standards.*

Following your valuable suggestions, we have incorporated relevant references from the international literature into the sections concerning cut-off values, factor analysis, and expert selection. These additions can be found in lines 144, 258–260, 268–272, and 293.

*2- The explanation of the relationship between the 10 extracted factors and the original four dimensions requires clearer conceptual justification to ensure theoretical coherence.*

We have further clarified the relationships between the four main dimensions and the ten extracted factors in lines 116–121 and 357–364. These ten factors represent lower-order constructs derived from the original four dimensions. Additionally, to present these relationships more intuitively, we have added Figure 5.

*3- Regarding data availability, while your justification is understood, please include a formal* *data availability statement in the manuscript clarifying access conditions.*

We appreciate you bringing this important point to our attention. In response, we have now added a formal data availability statement, which can be found on line 558.

*4-* *Minor language improvements were mentioned; however, I notice some problems in whole manuscript. Please make sure that all are fine.*

We have carefully implemented the revisions you requested, including adjustments to grammar, formatting, and wording.

*5- Please confirm that all ethical considerations (anonymity, voluntariness, and consent procedures) are explicitly and clearly stated within the Methods section.*

Thank you very much for raising this important issue. In response, we have now clearly outlined the relevant information regarding “anonymity, voluntariness, and consent procedures” in lines 128–130.

*6-Finally, I would like to see a Figure in AMOS program with all items and subdminesions.*
 As you recommended, we have now added a complete AMOS model diagra（Figure 5） to better illustrate the relationships among factors and items at each level, and its description begins at line 352.

**We greatly appreciate the constructive feedback you have provided throughout the review process. In the revised manuscript, we have carefully addressed all of the comments and concerns raised. We believe that the changes made have significantly strengthened the paper. Should any further revisions be required, please do not hesitate to let us know. Thank you again for your time and expertise.**
